# Menstrual Cycle Variation in MRI-Based Quantification of Intraluminal Gas in Women With and Without Dysmenorrhea

**DOI:** 10.3389/fpain.2022.720141

**Published:** 2022-05-11

**Authors:** Hyeyoung Oh, Eli D. Ehrenpreis, Frank F. Tu, Katlyn E. Dillane, Ellen F. Garrison, Nondas Leloudas, Pottumarthi V. Prasad, Kevin M. Hellman

**Affiliations:** ^1^Department of Obstetrics & Gynecology, North Shore University Health System, Evanston, IL, United States; ^2^Advocate Lutheran General Hospital, Department of Medicine, Evanston, IL, United States; ^3^Department of Obstetrics & Gynecology, University of Chicago, Pritzker School of Medicine, Chicago, IL, United States; ^4^Department of Radiology, North Shore University Health System, Evanston, IL, United States; ^5^Department of Radiology, University of Chicago, Pritzker School of Medicine, Chicago, IL, United States

**Keywords:** colonic gas, dysmenorrhea, irritable bowel syndrome, menstrual, pain, MRI

## Abstract

Women frequently report increased bloating, flatulence, and pain during the perimenstrual period. However, it is unknown whether women have more intraluminal gas during menses. To evaluate whether pain-free women or women with dysmenorrhea have different amounts of intraluminal bowel gas during the menses, we utilized magnetic resonance imaging (MRI) to determine colonic gas volumes throughout the menstrual cycle. To avoid dietary influence, the participants were instructed to avoid gas-producing foods before their scheduled MRI. We verified the measurement repeatability across the reviewers and obtained an intraclass correlation coefficient of 0.92. There were no significant differences in intraluminal gas volume between menses and non-menses scans (*p* = 0.679). Even among the women with dysmenorrhea, there was no significant difference in the intraluminal gas volume between menses and non-menses (*p* = 0.753). During menstruation, the participants with dysmenorrhea had less intraluminal gas than participants without dysmenorrhea (*p* = 0.044). However, the correlation between the bowel gas volume and the pain symptoms were not significant (*p* > 0.05). Although increased bowel symptoms and bloating are reported in the women with dysmenorrhea during menses, our results do not support the hypothesis that increased intraluminal gas is a contributing factor. Although dietary treatment has been shown in other studies to improve menstrual pain, the mechanism responsible for abdominal symptoms requires further investigation. Our findings demonstrate that the intraluminal bowel gas volume measurements are feasible and are unaffected by menses under a controlled diet. The method described might prove helpful in future mechanistic studies in clarifying the role of intraluminal bowel gas in other conditions.

## Introduction

One in every three reproductive-age women has abdominal pain during menses that limits routine activity (1). During the perimenstrual period, women also frequently report increased bloating and flatulence (2, 3). Bloating and excessive flatulence are often associated with the abdominal pain, possibly due to the distension from an increased intraluminal gas (4). However, it is unknown whether women have more intraluminal gas during menses. It is also unknown whether the women with dysmenorrhea have more intraluminal gas than those without dysmenorrhea.

Irritable bowel syndrome (IBS) is more commonly reported in women with dysmenorrhea. In addition, the symptoms of IBS can worsen during menses (5). The prior studies of patients with IBS have demonstrated that the excess gas production correlates with the perception of abdominal pain and can be partially resolved with a reduced Fermentable Oligo-, di-, Mono-saccharides And Polyols (FODMAP) diet (6). Although one study has shown that dietary treatment in women with IBS can improve menstrual pain (7), this study did not specifically examine gas volume changes. Although some studies have suggested that the bowel gas symptoms are correlated to measurements of the intraluminal bowel gas (8–11), other studies have not (12–14). In general, the evidence for an excess gas production in IBS or patients with excessive bloating is mixed and more research is needed [for review see Ref. (4)]. Therefore, the measurement of bowel gas could elucidate the role of excess intraluminal gas in menses-related abdominal symptoms and carries the potential for the development of new therapies in the women with dysmenorrhea.

This study utilized magnetic resonance imaging (MRI) to determine colonic gas volumes throughout the menstrual cycle. Our purpose was to objectively assess intraluminal gas volume changes occurring during menses in women with dysmenorrhea and to compare those changes to those occurring in pain-free control participants.

## Methods

We performed an institutional review board-approved secondary analysis of the data collected from prior and ongoing studies focused on uterine physiology (including ClinicalTrials.gov: NCT04145518) conforming with the Declaration of Helsinki. All participants signed a written consent form. After completing a screening questionnaire and menstrual diaries, the participants were divided into the following two groups: Those with dysmenorrhea and a pain-free controls. The dysmenorrhea group, (*n* = 49) had moderately painful dysmenorrhea (>5 on a 0–10 numerical rating scale). The pain-free control group (*n* = 15) had little menstrual pain (<2/10). All available scans from prior studies were used in this secondary analysis.

Based on the electronic diary data from participants, MRI examinations were arranged within the first 48 h after the start of menstrual bleeding. Most scans were scheduled in the morning between 9 a.m. and 11 a.m. An MRI examination was also scheduled during the peri-ovulatory phase of the study subject's menstrual cycle.

The previous studies have suggested that high intestinal gas volumes could interfere with uterine imaging (15). Therefore, we implemented a simplified version of the FODMAP diet in all patients (6). Participants were instructed to avoid the most common high gas-producing foods (e.g., beans, cabbage, cauliflower, dairy, and whole wheat) 24 h before their scheduled MRI. The participants were asked to avoid short-acting, over-the-counter analgesics (such as nonsteroidal anti-inflammatory drugs) as well as opioid medications for at least 6 h before the MRI scanning visit. The participants were also instructed to avoid longer-acting nonsteroidal anti-inflammatory drugs for 12 h before the MRI scan.

After confirming that the participants met safety criteria for the procedure, the images were obtained with a 3T MRI (Magnetom Verio, Siemens Healthcare, Erlangen, Germany) using a high-performance body coil. The anatomical sequences were first obtained with half-Fourier acquisition single-shot turbo spin echo imaging (HASTE) sequences). After about 5 min of anatomical scans, the sagittal gradient–echo sequences were obtained during a 12-s breath-hold (field of view: 300 mm × 300 mm, 5 slices, thickness: 5.0 mm, matrix: 256 × 256, repetition time: 50 ms, time to echo: 8 equally spaced between 3.09 and 32.3 ms). On average, it took 4 min to obtain these images. These gradient–echo sequences were used for the analyses of this study. Other sequences, beyond the scope of this study, were obtained afterward for the study of uterine physiology.

Intraluminal sigmoid colon gas was measured by quantifying the 2D area of susceptibility artifact from the R2^*^ map generated from gradient–echo sequences [similar to (15)]. The volume of intraluminal gas was measured with Firevoxel (of build 362 from Firevoxel.org) across all slices by selecting all intraluminal voxels with signal susceptibility artifact. We validated that bowel gas volume measurements were accurate using a 15 ml tube filled with methane inserted in a stuffed bovine large intestine (Romanian Kosher Sausage Co., Chicago, IL). All measurements were conducted by a single reviewer (HO) naïve to participant identity or group.

Stata 13.1 (College Station, Texas) was used for statistical analyses. To account for the within-subjects effects (menses *vs*. non-menses) and the between-subjects effects (pain-free controls *vs*. women with dysmenorrhea) and these associations with intraluminal gas, a linear mixed regression model (also known as a mixed effects model) was used. The linear mixed regression is an optimal solution for simultaneously obtaining both between-subjects effects (e.g., unpaired analyses) and within-subjects effects (e.g., paired analyses) when the data is partially missing (16). Some of the participants did not complete both scans (Table 1). However, we had more than 80% power to detect a medium effect size difference (*d* = 0.5, α = 0.05) in the volumes of bowel gas in women with dysmenorrhea compared to off-menses or pain-free controls using the mixed regression model. Therefore, we were adequately powered to compare the intraluminal gas in both groups during menses. The data for this study is available on open science framework, https://doi.org/10.17605/OSF.IO/VGE5R.

**Table 1:** Demographics and pain characteristics.

	**Pain-free controls**	**Dysmenorrhea**	***p***
*n*	15	49	
Age (years)	27 (20–37)	24 (20–34)	0.874
BMI (kg/m^2^)	22.3 (21.1–24.7)	24.2 (22.0–28.2)	0.048
**Race:**			
Caucasian	53.9%	57.7%	0.321
Asian	23.1%	17.3%	0.882
African–American	15.4%	25.0%	0.295
Other	7.7%	0.0%	0.071
Menstrual pain^1^	7 (3–12)	74 (59–82)	<0.001
Bowel pain^1^	3 (0–8)	20 (1–54)	0.005
*n* menses scans	14	48	
*n* non-menses scans	10	26	

*Results were reported as the median interquartile range (IQR) or percentage*.

1 *Menstrual and bowel pain were self-reported on a 0–100 visual analog scale (0: No pain, 100: Worst pain imaginable)*.

*Mann–Whitney or Chi-square tests when relevant were used to compare the demographic and pain variables*.

## Results

### Demographics

Table 1 shows a comparison of demographic factors and pain reports between the participants in the two groups. The demographic composition of both groups was comparable. However, the participants with dysmenorrhea reported significantly worse menstrual pain and pain during bowel movements than pain-free controls (Table 1, *p* < 0.01).

### Reproducibility of Intraluminal Gas Measurement

**Figure 1:**
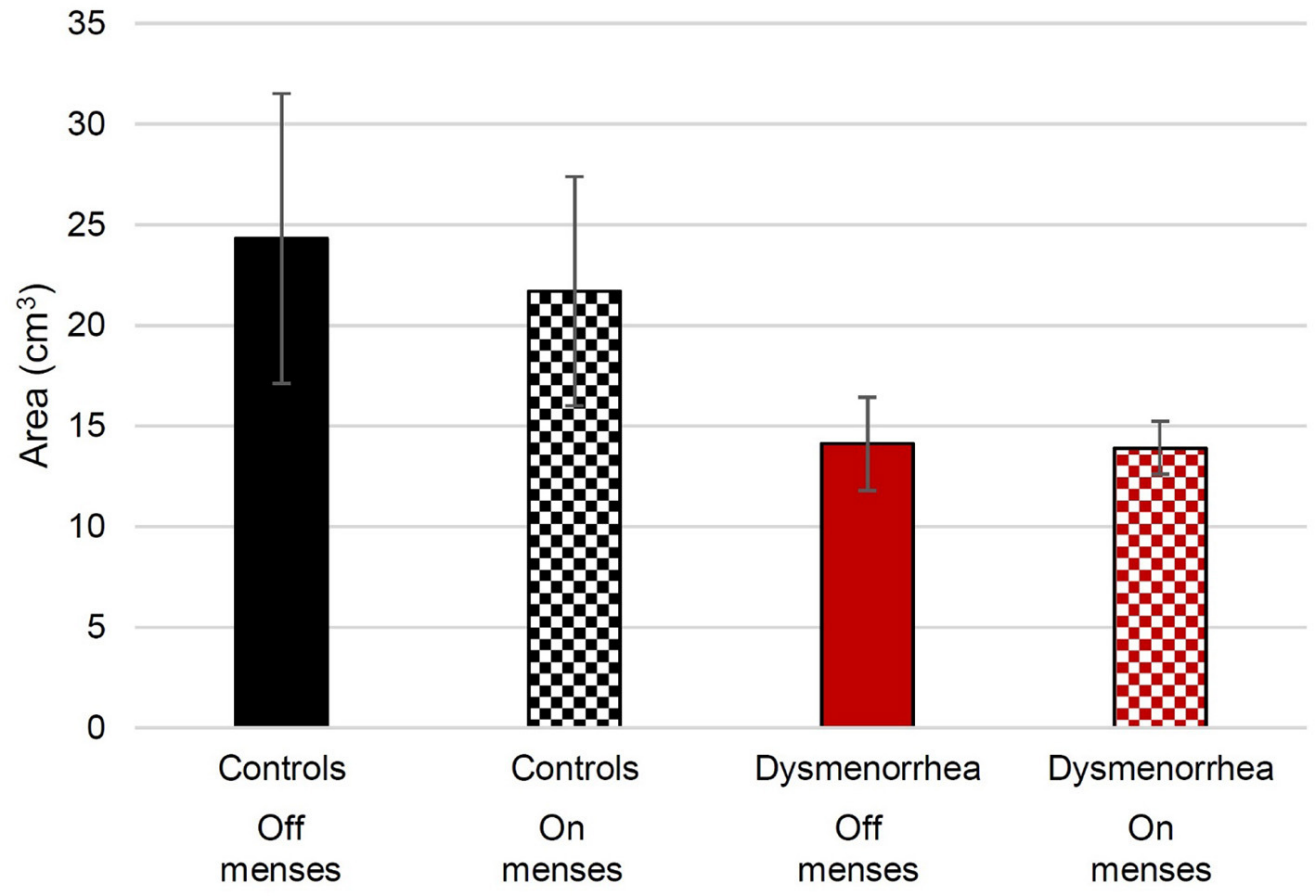
The average volume of measured intraluminal sigmoid colon gas in pain-free control participants and women with dysmenorrhea. The error bars show the standard error of the mean.

The volume of intraluminal gas was measured across participants by reviewers unaware of the menstrual status of the participants (Figure 1). We verified the reliability of this method by repeating measurements with another reviewer and obtained a two-way mixed-effects model with an absolute agreement, single rater, intraclass correlation coefficient of 0.92.

### Differences in Intraluminal Gas During Menses and the Peri-Ovulatory Phase

We hypothesized that if intraluminal gas contributed to menstrual symptoms, the participants would have more intraluminal gas during their menses than off-menses. Although the bowel gas was highest in the pain-free controls off-menses, there were no significant statistical differences (see Figure 1). The linear mixed effects regression was used to evaluate for differences between menses and non-menses. The difference between intraluminal gas during menses and non-menses across the groups (Figure 1) was not significant (*z* = −0.41; *p* = 0.679). The women with dysmenorrhea did not have more intraluminal gas during menses than those on non-menses visits (*z* = 0.31; *p* = 0.753).

### Differences in Intraluminal Gas Between Women With Dysmenorrhea and Pain-Free Controls

We also hypothesized that if intraluminal gas contributed to menstrual pain, women with dysmenorrhea would have more intraluminal gas than pain-free controls during menses. A linear mixed effects regression was used to evaluate whether women with dysmenorrhea have more bowel gas than pain-free controls. During menses, the participants with dysmenorrhea had less intraluminal gas than pain-free controls (*z* = −2.02; *p* = 0.044). Although a similar magnitude discrepancy was observed off-menses, the differences were not statistically significant (*z* = −1.84; *p* = 0.066).

### Correlations Between Intraluminal Gas and Pain Symptoms During Menses

Finally, we examined whether there were any significant correlations between the severity of pain symptoms and intraluminal gas during menses. The correlation between the menstrual pain (*r* = −0.16; *p* = 0.202) and the bowel pain (*r* = −0.19; *p* = 0.138) with intraluminal gas volume was not statistically significant.

## Discussion

These results demonstrate that while temporarily avoiding the high gas-producing food, women with dysmenorrhea do not have more intraluminal gas while menstruating than when not menstruating. Similarly, women with dysmenorrhea do not have more intraluminal gas compared to pain-free participants. Indeed, in our limited data set, on average, women with dysmenorrhea had less bowel gas than pain-free controls.

Although an increased bowel symptoms and bloating occur during menses and are present in women with dysmenorrhea (2, 3), our results do not support the hypothesis that increased the intraluminal gas contributes to these symptoms. It is possible that the decreased levels of intraluminal gas in women with dysmenorrhea could be due to alterations in their intestinal microbiome. Although the intestinal microbiome has not yet been well-characterized, the patients with severe dysmenorrhea had lower vaginal lactobacilli levels and higher abundance of Prevotella, Atopobium, and Gardnerella than patients with mild dysmenorrhea (17). Alternatively, women with dysmenorrhea may have increased visceral sensitivity. The decreased tolerance for intraluminal distension could lead to less tolerance of gas and result in expulsion. Patients with dysmenorrhea have been shown to have reduced distension thresholds for pain in the sigmoid colon, rectum, and bladder (18, 19). Future studies should investigate the relationship between the microbiome, visceral sensitivity, and intraluminal gas volumes.

Our study has several limitations. It remains possible that hormonally-induced dietary changes during menses could lead to increased gas. These changes would not be observable in our study since all subjects consumed a low gas diet before imaging. The previous studies have confirmed menstrual alterations in food cravings (20); these might have unknown effects on dietary content. Thus, the wider differences in intestinal gas could occur that were not detected in this study because of the restriction of gas-producing foods. Although one study has shown that dietary treatment in women with IBS can improve menstrual pain (7), the mechanisms responsible for improvement require future investigation.

Methodological limitations could have precluded our ability to detect the differences across the menses. However, another study using similar gradient–echo measurements suggested that MRI-based methods are valid for measuring bowel gas content (21). Specifically, this study demonstrated that the MRI measurements can detect an increased gas associated with the consumption of inulin, a naturally occurring, poorly absorbed fructan polysaccharide —that also increases symptoms associated with gas. In that study, the changes in bowel gas as measured by MRI were also paralleled by changes in breath hydrogen levels, further supporting the idea that MRI measurement of bowel gas is reflective of the fermentation of prebiotic substances. In our study, two different reviewers independently measured the volume of the measured gas. The reviewers achieved a high level of agreement, suggesting that the measurements are highly reproducible (single rater, intraclass correlation coefficient = 0.92). Other studies have similarly used the volumetric measurement of gas or distension (8). Nevertheless, it is possible that the other methods may provide better measurement of the bowel gas. Although the measurement of rectal methane may provide the best evidence for an abnormal fermentation in conditions like IBS (22), our technique is less invasive and holds promise to use in studies using interventions to improve symptoms. As this was a secondary analysis of a dataset with different established goals, the sample sizes were unequal. However, our choice of analytic methods and power analysis suggests that a medium effect size difference (*d* = 0.5) between the groups or across the menstrual cycle is unlikely.

In conclusion, our findings demonstrate that the intraluminal bowel gas volume measurements are feasible and have a high level of agreement across reviewers. However, while consuming a low gas (modified low FODMAP) diet, the intraluminal bowel gas is unaffected by menses. In fact, the participants with dysmenorrhea had less intraluminal gas than pain-free participants. The intraluminal bowel gas volume measurements might prove helpful in future studies focusing on physiologic effects of bowel gas.

## Data Availability Statement

The datasets presented in this study can be found in online repositories. The names of the repository/repositories and accession number(s) can be found below: open science framework, https://doi.org/10.17605/OSF.IO/VGE5R.

## Ethics Statement

The studies involving human participants were reviewed and approved by the NorthShore University HealthSystem Institutional Review Board. The patients/participants provided their written informed consent to participate in this study.

## Author Contributions

KH, FT, and PP conceived, designed, and obtained the funding for this project. EG, NL, and KD collected the data. HO, EE, and KH analyzed and interpreted the data. KH and EE drafted the manuscript. All authors read, edited, and approved the final manuscript.

## Funding

This study was funded by the Eunice Kennedy Shriver National Institute of Child Health and Human Development (HD081709, HD091502, and HD098193).

## Conflict of Interest

The authors declare that the research was conducted in the absence of any commercial or financial relationships that could be construed as a potential conflict of interest.

## Publisher's Note

All claims expressed in this article are solely those of the authors and do not necessarily represent those of their affiliated organizations, or those of the publisher, the editors and the reviewers. Any product that may be evaluated in this article, or claim that may be made by its manufacturer, is not guaranteed or endorsed by the publisher.
